# Learning Curve for Starting a Successful Single-Centre TAVR Programme with Multiple Devices: Early and Mid-Term Follow-Up

**DOI:** 10.3390/jcm13041088

**Published:** 2024-02-14

**Authors:** Balázs Magyari, Bálint Kittka, Ilona Goják, Kristóf Schönfeld, László Botond Szapáry, Mihály Simon, Rudolf Kiss, Andrea Bertalan, Edit Várady, András Gyimesi, István Szokodi, Iván Gábor Horváth

**Affiliations:** 1 Heart Institute, Medical School, University of Pécs, 7624 Pécs, Hungary; kittka.balint@pte.hu (B.K.); gojak.ilona@pte.hu (I.G.); schonfeld.kristof@pte.hu (K.S.); szapary.laszlo.botond@pte.hu (L.B.S.); simon.mihaly@pte.hu (M.S.); kiss.rudolf@pte.hu (R.K.); bertalan.andrea@pte.hu (A.B.); szokodi.istvan@pte.hu (I.S.); ivan.g.horvath@pte.hu (I.G.H.); 2 Szentágothai Research Centre, University of Pécs, 7624 Pécs, Hungary; 3 Department of Medical Imaging, Medical School, University of Pécs, 7624 Pécs, Hungary; varady.edit@pte.hu; 4 EconNet Research Group, Faculty of Business and Economics, University of Pécs, 7624 Pécs, Hungary; gyimesi.andras@ktk.pte.hu

**Keywords:** TAVR, learning curve, self-expandable transcatheter heart valve, balloon-expandable transcatheter heart valve, paravalvular leak, bicuspid aortic valve and radial paradox

## Abstract

**Aims:** We report 30-day, 1-year, and 3-year outcomes for a new TAVR programme that used five different transcatheter heart valve (THV) systems. **Methods:** From 2014 to 2020, 122 consecutive patients with severe aortic stenosis (AS) received TAVR based on the Heart Team decision. Outcomes were analysed for the whole study population and in addition the first 63 patients (Cohort A, 2014 to 2019) were compared to the last 59 patients (Cohort B, 2019 to 2020). Outcomes included VARC-2 definitions and device performance assessed via transthoracic echocardiography by independent high-volume investigators. **Results:** The mean patient age was 77.9 ± 6.1 years old, and 48 (39.3%) were male. The mean logistic Euroscore II was 4.2 ± 4.5, and the mean STS score was 6.9 ± 4.68. The systems used were as follows: Medtronic Corevalve Evolute R/PRO (82 patients—67.2%); Abbott Portico (13—10.6%); Boston Scientific Lotus (10—8.2%); Meril Myval (11—9%); and Boston Scientific Neo Accurate (6—5%). Access was transfemoral (95.9% of patients); surgical cut down (18% vs. percutaneous 77.8%); subclavian (*n* = 2); trans-axillary (*n* = 2); and direct aorta (*n* = 1). VARC-2 outcomes were as follows: device success rate 97.5%; stroke rate 1.6%; major vascular complication 3.3%; permanent pacemaker implantation 12.4%. At discharge, the incidences of grade I and II aortic regurgitation were 39.95 and 55.5%, respectively. At one year, all-cause mortality was 7.4% without admissions for valve-related dysfunction. The 3-year all-cause mortality and all-stroke rates were 22.9% and 4.1%, respectively. Between the 1-year and 3-year follow-ups, valve-related dysfunction was detected in three patients; one had THV system endocarditis that led to death. There was a remarkable but statistically non-significant decrease in mortality from Cohort A to Cohort B [four (6.3%) vs. one patient (1.7%), *p* = 0.195] and major vascular complications occurred at a significantly higher rate in the Cohort B [zero (0%) vs. four (6.8% patient, *p* = 0.036)]. Overall, we found that using multiple devices was safe and allowed for a learning team to achieve a high device success rate from the beginning (97.5%). **Conclusions:** TAVR with different THV systems showed acceptable early and mid-term outcomes for survival, technical success, and valve-related adverse events in high-risk patients with significant AS, even in the learning curve phase.

## 1. Introduction

For decades, surgical aortic valve replacement (SAVR) was the gold standard for patients with significant aortic valve stenosis (AS). However, a growing number of patients are unsuitable for open-heart surgery due to the ageing population. Transcatheter aortic valve replacement (TAVR) provides a possible cure for these patients. The indication for TAVR has been shifted from only high-risk patients [1,2] to intermediate- and low-risk patients. The PARTNER 2 trial proved that TAVR was similar to surgical aortic valve replacement for the primary endpoint of death or disabling stroke in intermediate-risk patients [3]. The Evolut low-risk trial tested the self-expanding CoreValve Evolut R and Evolut PRO Medtronic valves in a low-risk patient population; the 24-month estimated incidence of death or disabling stroke was similar in the TAVR and surgical arms, meeting the definition of statistical non-inferiority but not superiority [4]. The latest report of the 3-year outcomes of the Evolut low-risk trial confirmed the consistent benefit of TAVR over SAVR regarding all-cause mortality and disabling stroke, with significantly better hemodynamic valve performance [5]. The PARTNER 3 trial, however, reported that treatment with the balloon-expandable Sapien 3 transcatheter heart valve (THV) (Edwards Lifesciences) was superior compared to SAVR for the prevention of death, stroke, and rehospitalisation at one year, which was sustained at two years [6]. Moreover, no significant differences could be detected regarding the two composite primary outcomes in the most recently published 5-year follow-up of the PARTNER 3 trial [7]. Based on the cumulative available data, American guidelines recommend a shared decision-making process when choosing the type of aortic valve replacement (SAVR or TAVR) for patients aged between 65 and 80 [8]. On the other hand, European guideline recommend TAVR for older patients (>75 years), patients with high surgical risk, or those unsuitable for SAVR [9].

The learning curve of this technically complex TAVR procedure can vary [10,11], and there are no clear guidelines for organising a TAVR programme. Further, it is unclear whether it is safe for centres starting a new TAVR programme to use multiple TAVR systems. This study reports the 30-day, 1-year, and 3-year Valve Academic Research Consortium-2 (VARC-2) outcomes of the first 122 patients in our learning curve.

## 2. Methods

### 2.1. Study Design

This study examined the outcomes of the whole patient cohort and the difference in outcomes between an initial TAVR implantation learning period (Cohort A) and a higher-volume final year (Cohort B). This study is a single centre’s experience. Data were recorded in real time using our centralised, electronic, medical data-collecting system (e-MedSolution system) as part of standard care, and then analysed retrospectively. Data collection was approved by the Regional Research Ethics Committee (9435-PTE 2022).

### 2.2. Patient Population

From March 2014 to March 2020, 122 consecutive patients underwent a TAVR procedure at our institution (details below in baseline patient characteristics). The learning curve was analysed by comparing outcomes for the first 63 patients (Cohort A, 2014 March to 2019 March) to the last 59 patients (Cohort B, 2019 March to 2020 March). Cohort B was therefore conducted with a more experienced team (after completing Cohort A) and an optimal surgical volume (Cohort B was completed over one year; Cohort A was completed over five years). Thus, comparisons between these cohorts examine the effect of the volume–outcome relationship on the TAVR procedure. All TAVR procedures were paid for by the public healthcare system.

Included patients had high-gradient severe AS (valve area ≤ 1 cm^2^ or indexed valve area ≤ 0.6 cm^2^, Vmax ≥ 4 m/s, and mean aortic valve gradient ≥ 40 Hgmm). For patients with low-gradient AS (details below), severity and indication were based on dobutamine stress echocardiography and/or their native aortic valve computerized tomography (CT) calcium score. All patients had a New York Heart Association (NYHA) class of II or higher. Indication of the TAVR procedure was based on the Heart Team decision, including a vascular surgeon when an alternative access site should be used (trans-subclavian, trans-axillary). The operative risk was calculated using the logistic EuroSCORE and the STS score. The main exclusion criteria were acute myocardial infarction ≤ 14 days, left ventricular ejection fraction ≤ 20%, ongoing infection, hemodynamic instability, contraindication for antiplatelet and/or anticoagulant therapy, or life expectancy less than 12 months.

### 2.3. Device Description and Procedure

The Institute’s TAVR programme started in 2014 with the Lotus Valve System (Boston Scientific, Natick, MA, USA). After the recall of this THV, we switched to the Medtronic THV system (Medtronic, Minneapolis, MN, USA). From the first-generation Medtronic Corevalve THV, only 2 were implanted (in the Cohort A), followed by the second-generation Medtronic THV Evolut R (Cohort A-28 vs. Cohort B-23) and the latest-generation Evolut Pro System (Cohort A-19 vs. Cohort B-9). Due to some patients’ anatomic difficulties, we introduced three more THV systems: Portico (Abbott Vascular, Santa Clara, CA, USA), Accurate Neo (Boston Scientific, Marlborough, MA, USA), and Myval (Meril Life Sciences Pvt. Ltd., Vapi, India).

All TAVR procedures were performed in the dedicated hybrid operating room. Due to the importance of transoesophageal echocardiography guidance, general anaesthesia was used in most cases. We used local anaesthesia to decrease the risk of prolonged intensive care unit treatment in patients with severe pulmonary dysfunction. The standard access site was the femoral artery, if feasible. When this approach was unsuitable, alternative access sites included trans-subclavian, trans-axillary, or direct aortic access. Adjunct pharmacologic therapy included intraoperative ACT-guided heparin treatment. This was followed by dual antiplatelet therapy (aspirin 100 mg/day and clopidogrel 75 mg/day) for six months. If anticoagulant therapy was needed, clopidogrel (75 mg/day) with NOAC therapy was standard.

To achieve the best clinical outcomes possible, we set our TAVR programme as follows: The first operator (responsible for manipulating the THV systems after the guiding sheath is inserted) and the second operator (responsible for access site preparation in the case of percutaneous technique) stayed the same. Both operators started their interventional practice as transfemoral (TF) operators and were well-trained high-volume operators. For the percutaneous technique, the access site puncture was always ultrasound-guided. The vascular surgeon, the anaesthetist, and cardiac sonographers were the same throughout the study. The cardiac surgeon was in “stand-by” position during TAVR procedures (detailed data regarding the TAVR team are in Supplementary Table S1).

The TAVR programme selected each THV system to implant based on each patient’s anatomical characteristics. As a result, an uneven number of systems were implanted for each THV type. Thus, a direct comparison of these devices was outside the scope of this study. In the first 90 cases, we used two THV devices: 10 cases with Lotus and 80 cases with the Medtronic CoreValve system. The key non-device specific steps (puncture of the access site; introducing the large bore sheath; crossing the native valve; positioning the temporary pacemaker and stiff guidewire; performing balloon pre-dilatation, if needed) of the TAVR procedure were performed without a big variation in devices. The other 3 THV devices were introduced into our TAVR programme only thereafter. In every case, when a new THV device was used, based on the strict recommendation of the companies, the first cases were supervised by a high-volume proctor and collected to avoid sparse TAVR implantation with the new device. Supervised cases were performed in two separated occasions (3 cases in each), and the difference between them were 3 months with the Myval system, 2 weeks with the Portico system, and 2 months with the Acurate neo system.

### 2.4. Access Site

The preferred access site was transfemoral (TF) in most cases (95.9%). Initially, TF procedures were performed by surgical cut-down. After 22 (18%) successful TAVR implantations, the percutaneous technique became the standard method (77.8%). Surgical support was used for only dedicated, alternative vascular access procedures or in bailout events to solve complications (4%). One patient was implanted with direct aortic access. Detailed descriptions of TF, trans-subclavian, trans-axillary, and direct aortic approaches are in the Supplementary Materials.

### 2.5. Study Endpoints and Follow-Up

Safety and efficacy parameters were collected at discharge and 30-day, 1-year, and 3-year follow-ups. Safety was evaluated as a primary endpoint based on periprocedural outcomes. Short- and long-term hemodynamic performance were also primary endpoints and were based on transthoracic echocardiography by independent sonographers. As a secondary endpoint, the 30-day, 1-year, and 3-year combined safety endpoints were defined by VARC-2. The functional status of the patients was based on their NYHA functional class. All relevant endpoints were defined according to the VARC-2 definitions [12].

The severity of perioperative aortic regurgitation was evaluated by intraoperative echocardiography (transoesophageal or transthoracic echocardiography), angiography, and measurement of the aortic regurgitation index, as described previously [13].

### 2.6. Statistical Analysis

Descriptive statistics were used for continuous variables and expressed as mean ± standard deviation. Categorical variables are presented as number and percentage. The comparison between groups was performed using the Student’s *t*-test for categorical variables and *z*-test for proportions. All tests were two-sided at the 0.05 significance level. All calculations were performed using SPSS statistics (version 28.0.0.0, IBM, Armonk, NY, USA).

## 3. Results

### 3.1. Baseline Patient Characteristics

Between March 2014 and March 2020, 122 consecutive patients underwent TAVR at our institution. All patients had severe symptomatic AS based on echocardiographic measurements. Based on the latest European Society of Cardiology guideline for the management of valvular heart disease [14], most of the patients (86.9%) had high-gradient AS, 11.5% had low-flow, low-gradient AS (LFLG-AS), and 1.6% had paradoxical low-flow, low-gradient AS (PLFLG-AS). In patients with LFLG-AS and PLFLG-AS, the indication for aortic valve replacement was based on stress echocardiography [15,16] and/or the native aortic valve calcium score [17,18,19].

Among the patients, seven had a bicuspid aortic valve (5.7%), two (1.6%) had previously undergone mitral valve replacement with a mechanical prosthesis, one underwent urgent valve-in-valve (ViV) implantation for failed Corevalve Evolut valve implantation (details below), and two (1.6%) had elective ViV procedures for significant stenosis of the previously surgically implanted aortic bioprosthesis.

Due to a slow accumulation of implantation numbers, we reached the mandatory annual minimum of 50 TAVR procedures [20,21] only in the last year of this study (Cohort B). There were no significant differences between Cohort A and B in baseline characteristics, suggesting no influence of cohort characteristics on the outcomes. The baseline clinical and echocardiographic characteristics of the study population are shown in Table 1 and Table 2.

### 3.2. Procedural Outcomes

All relevant data regarding procedural and postprocedural parameters are presented in Table 3 and Table 4. There was a significant decrease in aortic peak (*p* = 0.007) and aortic mean gradient (*p* = 0.001) after the procedure. There were no procedural deaths, but five fatal outcomes during the hospitalisation period occurred. Detailed data regarding the reasons for in-hospital mortality are shown in Supplementary Table S2.

There was no periprocedural myocardial infarction due to coronary obstruction. In one patient, embolization caused amaurosis fugax that resolved by the one-year follow-up. Stroke was observed in two patients (1.6%). The first of these patients suffered from severe haemorrhagic stroke leading to death. The second patient had embolization into the arteria cerebri media. After fibrinolytic therapy, neurological symptoms improved, and the patient completed a successful neurologic rehabilitation programme.

There were no significant impairments of renal function during the hospitalisation period except for those three patients (0.24%) who were classified as stage 3 per the Acute Kidney Injury Network and died due to multi-organ failure. During the follow-up period, one patient started regular, intermittent renal replacement therapy. Renal function improved in 73 cases, possibly due to improved cardiac output that resulted in better renal blood flow.

Vascular complications were detected in 18 patients; 4 were major, and 14 were minor, according to the VARC-2 criteria (Supplementary Table S3). Vascular complications occurred in two types of situations. The first was the failure of a percutaneous closure device (two Proglide technique) (*n* = 5 complications/100 who received percutaneous closure). The second was in the trans-subclavian approach (*n* = 1 complications/2 who received trans-subclavian approach), where after control angiogram an endovascular treatment (balloon angioplasty with stent implantation) was mandatory to achieve patent flow at the donor artery. Major vascular complications were significantly higher in Cohort B [A: zero patients (0%) vs. B: four (6.8%), *p* = 0.036].

Other than vascular complications, there were no other significant differences between Cohorts A and B for device success. However, there was a remarkable but statistically non-significant decrease in in-hospital mortality [Cohort A: four patients (6.3%) vs. Cohort B: one (1.7%), *p* = 0.195]. All postprocedural outcomes (<72 h after the index procedure) are shown in Table 4.

#### Device Success and Re-Intervention

Device success was achieved in all but three patients (97.5%). In the first case, after the pericardiac tamponade was surgically corrected, TAVR was completed, but severe aortic regurgitation was observed. Immediate ViV was not performed. In the second case, the first Acurate neo valve dived into the left ventricle, causing cardiogenic shock. Immediate ViV was performed successfully with a new Acurate neo valve, and the patient’s hemodynamic parameters normalised. The third patient had a true bicuspid aortic valve (Type 0). Despite several balloon pre-dilatations and multiple manoeuvres, the Medtronic Evolute Pro 29 system could not cross the native aortic valve. For safety reasons and to avoid fatal complications (e.g., aortic rupture, rupture of the free wall), the TAVR procedure was terminated. One week later, the TAVR procedure was performed successfully using the Myval steerable delivery system.

### 3.3. VARC-2 Outcomes at Follow-Ups

#### 3.3.1. VARC-2 Outcomes at 30 Days

After the five in-hospital deaths (above), there were no subsequent deaths in the first 30 days; thus, 30-day all-cause mortality was 4.1%. There were no new strokes in the 30-day follow-up period after patient discharge. Irreversible renal injury (Acute Kidney Injury Network’s stage 3 classification) occurred in one patient, leading to intermittent, regular renal replacement therapy. Two patients received a repeat TAVR procedure due to severe valve-related dysfunction. In the first patient (described above), a second TAVR procedure was performed three days after the first implantation because severe aortic regurgitation was detected. The second patient had acute ViV implantation during the index procedure; after three days, repeat fluoroscopy was performed, and there was no additional migration of the first TAVR valve. This patient was readmitted due to cardiogenic shock after one week. Transoesophageal ultrasound detected further migration of the first TAVR valve, which led to severe aortic regurgitation and severe mitral stenosis. After readmission, acute SAVR and mitral valve replacement were performed successfully.

#### 3.3.2. VARC-2 Outcomes at 1-Year

Between the 30-day and 1-year follow-up, four additional deaths occurred: two due to a cardiac event, one due to the progression of ischaemic stroke that started immediately after the procedure, and one due to an infection that led to multiple organ failure. The mortality rate at one year was 7.4% (9/122) all-cause and 4.9% for cardiac mortality. In addition to the two in-hospital-evolving strokes, one other patient had a non-disabling ischaemic stroke, which resulted in a 2.5% (3/122) one-year all-stroke rate. After 30 days, there were no new hospital admissions for heart failure progression. One patient was at NYHA stage III. Five patients had valve-related dysfunction due to an elevated mean gradient on the THV (based on echocardiography) but did not need prosthetic valve re-intervention.

#### 3.3.3. VARC-2 Outcomes at 3 Years

**Figure 1:**
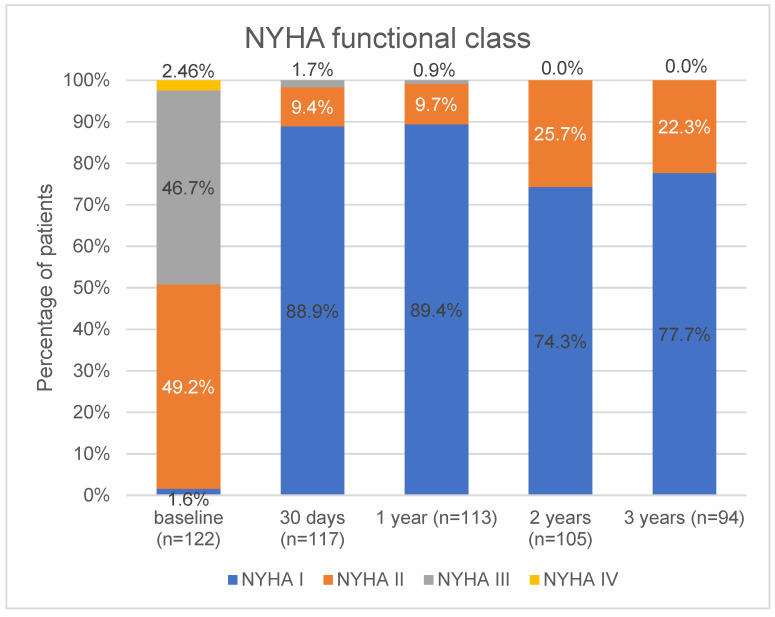
Baseline and follow-up clinical status of the patients based on classification by the New York Heart Association. Values are n (%).

At the three-year follow-up, 17 additional deaths had occurred. Two were due to a cardiac event. Therefore, the three-year mortality rate was 22.9% (28/122), with a 6.5% cardiac mortality rate (8/122). After the one-year follow-up, one non-disabling ischaemic stroke and one transient ischemic attack occurred, leading to a 4.1% (5/122) all-stroke rate at three years. After the TAVR procedure, the functional status of the patients improved. A minority were NYHA III, and none were NYHA IV. This finding was stable at the one-year follow-up. At the two-year follow-up, the number with NYHA I decreased significantly [113 (89.4%) vs. 105 (74.3%), *p* = 0.003], but the rest were at NYHA II with no significant changes at the three-year follow-up. Between the one-year and three-year follow-ups, four patients required hospitalisations for worsening heart failure. At the two- and three-year follow-ups, no patients had NYHA stage III or above (Figure 1). Valve-related dysfunction was detected in three additional patients. Two of them had an elevated mean gradient on the THV system without a need for re-intervention of the prosthetic valve. The third patient had THV system endocarditis, leading to their death. During the study period, at the 3-year follow-up, there were six patients who were lost to follow-up. These patients had visits via phone; therefore, echocardiographic data are missing regarding these patients.

Nine patients already had a permanent pacemaker implantation (PPI), and fourteen patients were implanted after the TAVR procedure; therefore, the new PPI rate was 12.4% (14/113). There were no significant differences between patients with or without the need for PPI for implantation depth, age, Euroscore, Euroscore II, STS score, aortic valve calcium score, or presence of calcium in the left ventricular outflow tract. Detailed data are shown in Table 5. At the one-year follow-up, one patient had implantable cardioverter defibrillator pacemaker implantation. Between the one- and three-year follow-ups, one patient had PPI.

**Figure 2:**
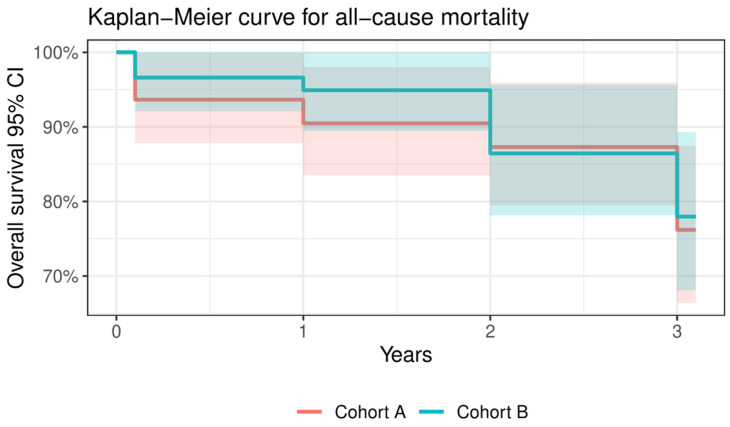
Kaplan–Meier curve for all-cause mortality regarding Cohort A and Cohort B.

**Figure 3:**
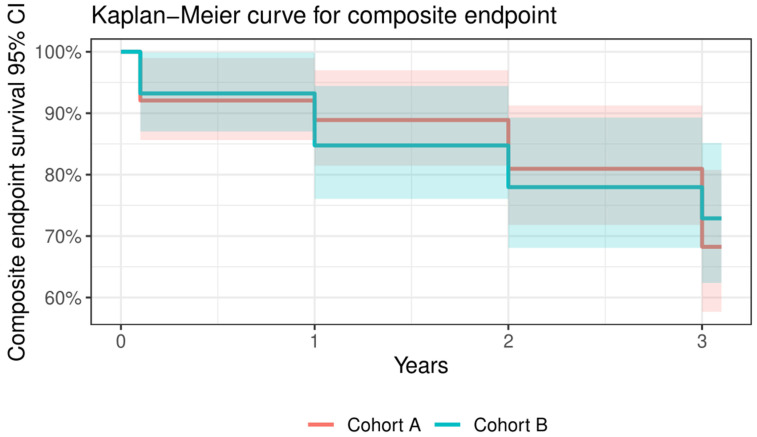
Kaplan–Meier curve for composite endpoint regarding Cohort A and Cohort B. Composite endpoint included cardiac mortality, all stroke, and valve-related dysfunction.

**Figure 4:**
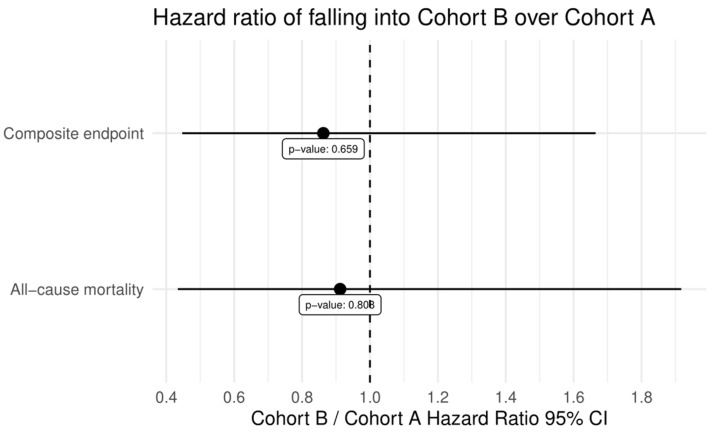
Hazard ratios comparing Cohort A and Cohort B regarding all-cause mortality and composite endpoint.

There was a significantly higher rate of PPI during the index procedure in Cohort B [A: two patients (3.2%) vs. B: twelve (20.3%), *p* = 0.002]. There were no other significant changes between Cohorts A and B in VARC-2 definitions at any time point of the follow-up period except for the major vascular complication (details above). All relevant data from 30-day, 1-year, and 3-year outcomes of the whole cohort and in the subgroups are summarised in Table 6 and Figure 2, Figure 3 and Figure 4.

### 3.4. Echocardiographic Outcomes and Valve Durability

The peak aortic gradient decreased significantly after the procedure without further significant changes between the time periods, including discharge vs. 30-day follow-up; 30-day follow-up vs. 1-year follow-up; 1-year follow-up vs. 2-year follow-up; and 2-year follow-up vs. 3-year follow-up. The same results could be detected regarding the mean aortic gradient. Global ejection fraction was stable during the examined period.

There were no significant differences between Cohorts A and B for peak aortic gradient, mean aortic gradient, and global ejection fraction at any point of the follow-up period. Nevertheless, at the one-year follow-up, the global ejection fraction was statistically higher in Cohort B. This finding has no relevance from a clinical point of view. Details are shown in Table 7.

An aortic regurgitation grade of two or above was detected in a minority of the patients and did not require THV reintervention based on the patients’ clinical condition. Moreover, no relevant paravalvular leaks were detected throughout the follow-up period. Echocardiography found a significant decrease in the number of patients with mitral regurgitation grade III or IV, with a slight increase after one year. This finding supports the theory that in the presence of severe aortic stenosis, most mitral regurgitations are secondary to severe left ventricular pressure overload caused by the AS; therefore, severe mitral regurgitation should not be an absolute exclusion criterion in patient selection.

**Figure 5:**
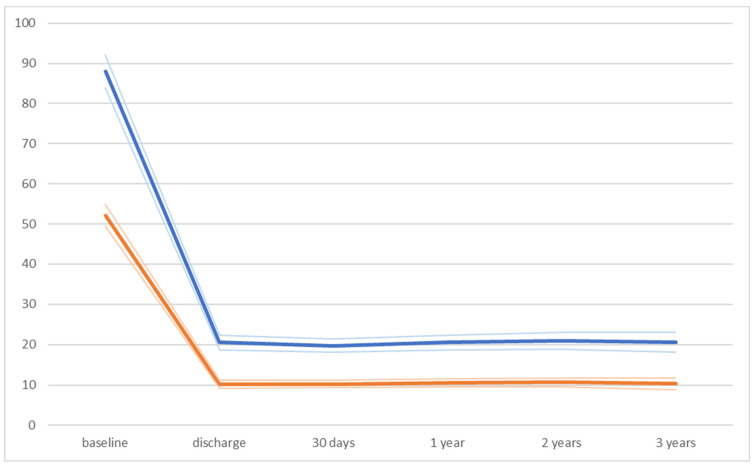
Data regarding aortic peak gradient (blue line) and aortic mean gradient (orange line) in the whole study population.

**Figure 6:**
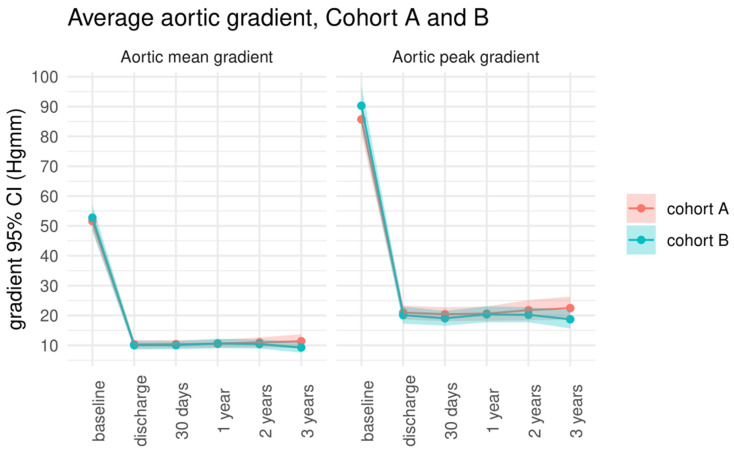
Comparison between Cohort A and Cohort B regarding aortic mean gradient and aortic peak gradient.

All relevant data from echocardiographic measurements are listed in Table 8 and Figure 5 and Figure 6.

## 4. Discussion

We report a high device success rate (97.5%), which is outstanding for a learning phase with five different THV systems, especially when compared to the learning curve for TAVR in high-volume studies [22,23]. The rates of mortality, stroke, and vascular complication were at least comparable to other studies, even when comparing our results with higher-annual-implanting centres [23,24,25]. Moreover, most studies analysing the learning curve focus on the procedural and 30-day results, and long-term data are sparce. Thorough planning was crucial to achieving these results. Complications were also minimised by analysing CT measurements to choose the appropriate TAVR system for the patients’ anatomical features [26]. The standardization of TAVR procedures (well-trained staff, fixed team members) may have been responsible for our high procedural success with a low complication rate. One of the operators had expertise in peripheral arterial intervention in case of complications at the access site. We used transoesophageal ultrasound guidance, which can help optimise the THV implantation depth and give precise information about paravalvular leaks. We also did not have patients with coronary obstruction, a condition with high in-hospital and one-year mortality rates. Finally, we used the Myval or Acurate neo systems in cases of low coronary take-off because their technical advantages avoid a fatal clinical scenario.

As mentioned, we reached the mandatory annual minimum of 50 TAVR procedures [20,21] only in the last year of this study (Cohort B). Our mortality rate decreased remarkably in this last year, but this difference did not reach statistical significance; this is likely due to the low case numbers. Nevertheless, this decrease in in-hospital mortality from the low-volume Cohort A to the optimal-volume Cohort B could confirm the volume–outcome relationship for the TAVR procedure described by Keier [27]. Our mortality rate is in the expected range [22,23].

Our vascular complication rate is comparable to other studies [22,23]. There was a significantly higher rate of major vascular complications in the last year (Cohort B). This could be explained by more complicated patients in Cohort B than in Cohort A. Further, one-third of the patients in Cohort A were implanted from surgical cut-down; that may have been protective against vascular complications. It should be emphasised that no deaths were due to vascular complications in Cohort B; therefore, this higher rate of major vascular complications in Cohort B did not negatively influence patient survival. In order to minimize vascular access site complication, ultrasound-guided access site puncture is mandatory, especially in “by default radial operators”. Finally, there were no significant differences in valve durability between the first 63 patients (Cohort A) and the last 59 patients (Cohort B). This suggests that THV system training alone may provide stable hemodynamic performance of the prosthetic valve from the beginning.

There were seven patients with a bicuspid aortic valve (5.7%). Performing TAVR procedures in patients with bicuspid aortic valves can raise several difficulties. Crossing the native valve with the delivery systems can be challenging (especially in Type 0 form). In these cases, standard stiff wires (SAFARI for Boston Scientific, Confida Guidewire for Medtronic) can be changed for stiffer wires (Lunderquist or Lunderquist double curve for Cook Medical) to achieve adequate support for delivering the valve, but this increases the chance of traumatic injury to the left ventricle. Steerable delivery systems are superior for overcoming this anatomic situation compared to standard non-steerable delivery systems using standard guidewires. Paravalvular leakage is another problem for a bicuspid aortic valve. With the higher radial force of the balloon-expandable THV, we can circularise the native anulus, thereby minimising the potential sites for a paravalvular leak. Newer-generation self-expandable valve systems with adaptive sealing mechanisms are designed to solve this problem [28]. Using the balloon-expandable THV system in BAV anatomy may help to overcome those difficulties which come from the anatomical differences and, therefore, may simplify the TAVR procedure in this clinical scenario. However, our small case number is a limitation; TAVR procedures were performed in patients with BAV anatomy successfully, and the technical advantage could be confirmed even in our learning curve.

As mentioned, there was a significantly higher rate of PPI during the index procedure in Cohort B [A: two patients (3.2%) vs. B: twelve (20.3%), *p* = 0.002]. The PPI rate was well within the expected range, a positive outcome for a learning team. The MIDAS strategy (MInimizing Depth According to the membranous Septum [29]) states that minimising implantation depth can decrease the rate of PPI. This approach may have kept our PPI rate in the expected range. Overall, there were no significant differences between patients with or without PPI regarding any known risk factors, so this complication was not procedure-related and was rather patient-related. Due to the uneven implantation number in the different THV systems, the comparison between different THV systems regarding PPI was out of the scope of this report.

Elevated serum creatinine levels can lead to higher perioperative mortality risk. We did not see this relationship in our patient cohort. The reason for this phenomenon could be the improvement in cardiac output after the TAVR procedure that eliminated the severe left ventricular afterload.

This study reported our learning curve with the TAVR procedure. Our use of several different THV systems and a relatively low implantation number might be a limitation; however, it allowed for the selection of the most appropriate THV system for the patients’ anatomical properties to achieve the best clinical outcomes. Being aware of the pros and cons of the different THV systems provided the flexibility of choice so that an operator could change between different devices more easily and not try to address different patient anatomies with a single THV system. This approach did not appear to affect the device success rate.

## 5. Conclusions

There are no clear recommendations for centres starting TAVR programmes, particularly regarding which THV system to start with and the safety of using different THV systems while learning. Our results suggest that learning with multiple THV systems was safe when a well-trained team followed the instruction rigorously. During the learning curve, we found no differences between initial, low-volume TAVR (Cohort A) and later, high-volume TAVR (Cohort B) for mortality, although there appeared to be a non-significant decrease in mortality with experience. Using constant roles in the TAVI team, especially in the learning curve, operator-related complication rate is reducible. Overall, a well-trained interventional team highly adhering to the instruction for use of the different THV systems with ultrasound-guided access site puncture can reach a high device success rate with good short- and long-term results from the beginning. Regarding our results, THV device selection based on the local cost and device availability is safe and effective for starting a TAVR programme.

## Figures and Tables

**Table 1:** Baseline characteristics of the whole population in the two cohorts and the comparison of the Cohort A and Cohort B. BAV: balloon aorto-valvuloplasty. MVR: mitral valve replacement. AVR: aortic valve replacement. NA: not added value.

Baseline Characteristic of Study Population				
	Overall (*n* = 122)	Cohort A (First 63 Patients)	Cohort B (Last 1 Year, *n* = 59)	*p* Value
Age (yrs)	77.9 ± 6.1	78.7 ± 5.6	77.6 ± 6.6	0.325
Men	48	24	24	0.77
Body mass index (kg/m^2^)	29.3 ± 4.8	28.7 ± 4.5	29.9 ± 5.1	0.175
Body surface area (m^2^)	1.87 ± 0.20	1.86 ± 0.2	1.87 ± 0.21	0.834
Hypertension	113	60	56	0.934
Diabetes mellitus	32	19	13	0.308
Hyperlipidemia	87	43	43	0.575
NYHA class III or IV	60	28	32	0.28
Ischaemic Heart Disease	56	29	26	0.828
Prior Myocardial Infarction	17	7	10	0.352
Prior PCI	40	21	19	0.894
Prior CABG	15	9	6	0.489
Peripheral artery disease	21	4	5	0.654
Cerebrovascular disease	10	9	2	0.036
Pulmonary disease	15	6	9	0.335
Previous BAV	21	14	6	0.066
Permanent PM	9	6	3	0.349
Atrial fibrillation	25	15	10	0.348
Logistic EuroSCORE	15.9 ± 14.6	16.6 ± 12.8	15.2 ± 13.6	0.554
Logistic EuroSCORE II	4.2 ± 4.5	4.7 ± 4.2	4.3 ± 4.8	0.667
STS score (%)	6.9 ± 4.68	7.2 ± 3.6	6.7 ± 5	0.497
Serum creatinine (μmol/L)	100.3 ± 46.4	99.9 ± 42.2	100.7 ± 50.8	0.936
Estimated GFR (mL/min)	60.5 ± 24.2	59.6 ± 26.6	61.4 ± 21.6	0.678
Estimated GFR < 60 mL/min	70	40	28	0.075
Bicuspid valve	7	3	4	0.632
Prior MVR	2	0	2	0.141
Prior AVR	1	0	1	0.299
Dialysis	0	0	0	NA
Procedure status				
elective	114	61	53	0.119
urgent	8	2	6	0.119
acute	0	0	0	NA

**Table 2:** Baseline parameters of transthoracic echocardiography in the study population and in the subgroups of high-gradient AS; low-flow, low-gradient AS, and paradox low-flow, low-gradient AS.

Echocardiographic Parameters of the Study Population (*n* = 122)	
**Mean LVEF**	55.9 ± 10.6
Mean AoVmax (m/s)	4.62 ± 0.62
Aortic peak gradient (Hgmm)	87.6 ± 22.7
Aortic mean gradient (Hgmm)	52.7 ± 15.9
Mitral insufficiency III or IV	21
Tricuspid insufficiency III or IV	19
sPAP ≥ 60 Hgmm	9
***High-gradient AS***	*n* = 106 (86.9%)
Mean LVEF	57.6 ± 6.3
Mean AoVmax (m/s)	4.7 ± 0.6
Aortic peak gradient (Hgmm)	90.2 ± 22.6
Aortic mean gradient (Hgmm)	53.8 ± 16
***Low-flow, low-gradient AS***	*n* = 14 (11.5%)
Mean LVEF	31.3 ± 5.5
Mean AoVmax (m/s)	3.91 ± 0.61
Aortic peak gradient (Hgmm)	67.8 ± 19
Aortic mean gradient (Hgmm)	37.5 ± 9.5
***Paradox low-flow, low-gradient AS***	*n* = 2 (1.6%)
Mean LVEF	62.5 ± 9.2
Mean AoVmax (m/s)	3.84 ± 0.34
Aortic peak gradient (Hgmm)	59 ± 11.3
Aortic mean gradient (Hgmm)	34.5 ± 4.9

**Table 3:** Detailed data of invasive examination in the whole study population and in the subgroups using different transcatheter heart valve systems. ARI: aortic regurgitation index.

*Variable*	*Overall* *(n = 122)*	*Medtronic Corevalve* *(n = 82, 67.2%)*	*Portico* *(n = 13, 10.6%)*	*Myval* *(n = 11, 9%)*	*Acurate-Neo (n = 6, 8.2%)*	*Lotus* *(n = 10, 8.2%)*
**Type of anesthesia**						
general	118	81	10	11	6	10
local	4	1	3	0	0	0
**Access site**						
femoral	117	78	13	11	6	10
subclavia	2	2	0	0	0	0
axillaris	2	2	0	0	0	0
direct aortic	1	1	0	0	0	0
Contrast agent	154 ± 119	144.5 ± 125.6	193.1 ± 98.3	244 ± 98	158.6 ± 76.7	91.7 ± 72.6
Operation duration (min)	89.6 ± 38.6	91.1 ± 40.9	85.1 ± 36.1	91.4 ± 41.1	69 ± 14.4	100.1 ± 32.4
Predilatation	39	10	13	10	6	0
Postdilatation	17	9	3	1	2	2
Preimpl. peak AV gradient	91.8 ± 24.7	86.5 ± 21.3	103.9 ± 36.4	96.5 ± 28.5	95.5 ± 16.2	99.3 ± 21.7
Preimpl. mean AV gradient	53.01 ± 14.9	52.36 ± 12.1	56.9 ± 21.1	56.7 ± 16.8	53.5 ± 12.5	58.4 ± 10.3
Postimpl. peak AV gradient	34.2 ± 9.6	33.6 ± 9.4	38.5 ± 8.1	34.3 ± 11.1	42.3 ± 4.1	28.6 ± 11.5
Postimpl. mean AV gradient	11.9 ± 6.4	12.5 ± 6.1	12.2 ± 4.4	8.4 ± 9.1	9.2 ± 7.5	13.9 ± 6.5
ARI	26.2 ± 9.8	26.3 ± 9.8	23.6 ± 9.6	27.2 ± 11.1	23 ± 4.7	30.7 ± 11.3
Permanent PM impl.	14 (12.4%)	10 (12.1%)	2 (15.3%)	1 (9.1%)	0	1 (10%)

**Table 4:** Detailed data of postprocedural outcomes (<72 h after the index procedure) of the whole study population and in Cohort A and Cohort B, based on VARC-2 definition.

Postprocedural Outcomes <72 h after the Index Procedure				
Outcome	Overall (*n* = 122)	Cohort A (First 63 Patients)	Cohort B (Last 1 Year, *n* = 59)	*p* Value
	No. (%) of events			
In-hospital mortality	5 (4.1%)	4 (6.3%)	1 (1.7%)	0.195
Device success	119 (97.5%)	62 (98.4%)	58 (98.3%)	0.963
Myocardial infarction	0 (0%)	0 (0%)	0 (0%)	-
Coronary obstruction	0 (0%)	0 (0%)	0 (0%)	-
Stroke or TIA	3 (2.4%)	2 (3.2%)	1 (1.7%)	0.598
Acute kidney injure, stage 2 or 3	3 (2.4%)	3 (4.7%)	0 (0%)	0.09
Major vascular complications	4 (3.3%)	0 (0%)	4 (6.8%)	0.036
Cardiac tamponade	0 (0%)	1 (0%)	1 (0%)	0.963
Annulus rupture	0 (0%)	0 (0%)	0 (0%)	-
Valve malpositioning	2 (1.6%)	1 (1.6%)	1 (1.7%)	0.963
Need for a second valve	2 (1.6%)	1 (1.6%)	1 (1.7%)	0.963
Posptocedural AR grade III or IV	1 (0.9%)	1 (0.9%)	0 (0%)	0.331

**Table 5:** Detailed data of comparison between patients with and without permanent pacemaker implantation. Ca score: Agatston calcium score of the aortic valve based on CT examination. Ca in LVOT: existence of calcium nodulus in the left ventricle outflow tract based on CT examination.

	Non Pm (*n* = 108, 88.5%)	PM (*n* = 14, 12.5%)	*p* Value
Age	78.6 ± 5.8	76.3 ± 7.6	0.232
Euroscore	15.8 ± 13.3	16.2 ± 12.9	0.933
Euroscore II	4.4 ± 4.5	5.02 ± 4.5	0.650
STS score	6.8 ± 3.8	8.1 ± 7.3	0.523
Ca score	2888 ± 1866	2767 ± 1108	0.822
Ca in LVOT	20	5	0.149
THV implantation depth			
Left coronary side (mm)	7.67 ± 2.9	7.99 ± 3.01	0.695
Non coronary side (mm)	7.6 ± 2.6	8.0 ± 3.6	0.584
Right coronary side (mm)	7.67 ± 2.6	8.23 ± 3.2	0.472
Average implantation depth (mm)	7.7 ± 2.6	8.1 ± 3.2	0.568

**Table 6:** Detailed data of postprocedural outcomes at 30-day, 1-year, and 3-year follow-ups of the whole study population and in Cohort A and Cohort B, based on VARC-2 definition.

*VARC-2 Outcomes at 30-Day, 1-Year, and 3-Year Follow-Ups*				
Outcome	Overall (*n* = 122)	Cohort A (First 63 Patients)	Cohort B (Last 1 Year, *n* = 59)	*p* Value
**30 days cumulative clinical outcomes (*n* = 122)**				
All-cause mortality	5 (4.1%)	4 (6.3%)	1 (1.7%)	0.195
Cardiac mortality	4 (3.3%)	3 (4.7%)	1 (1.7%)	0.341
All stroke	2 (1.6%)	1 (1.6%)	1 (1.7%)	0.962
Life-threatening bleeding	0 (0%)	0 (0%)	0 (0%)	-
Acute kidney injury, stage 2 or 3	1 (0.8%)	0 (0%)	1 (1.7%)	0.299
Coronary artery obstruction	0 (0%)	0 (0%)	0 (0%)	-
Major vascular complication	4 (3.3%)	0 (0%)	4 (6.8%)	0.035
New pacemaker implantation	14 (12.4%)	2 (3.2%)	12 (20.3%)	0.002
Valve-related dysfunction requiring repeat procedure (BAV, TAVI, or SAVR)	2 (1.6%)	1 (1.6%)	1 (1.7%)	0.962
**One-year cumulative clinical outcomes (*n* = 113, 92.6%)**				
All-cause mortality	9	6	3	0.348
Cardiac mortality	6	4	2	0.450
All stroke	3	1	2	0.520
Requiring hospitalizations for worsening heart failure	0	0 (0%)	0 (0%)	-
NYHA class III or IV	1	0	1	0.299
Valve-related dysfunction	5	1	4	0.148
**Three-year cumulative clinical outcomes (*n* = 94, 77.0%)**				
All-cause mortality	28	15	13	0.815
Cardiac mortality	8	5	3	0.524
All stroke	5	3	2	0.702
Requiring hospitalizations for worsening heart failure	4	1	3	0.278
NYHA class III or IV	0	0	0	-
Valve-related dysfunction	3	2	1	0.597

**Table 7:** Detailed data of comparison between Cohort A and Cohort B regarding peak aortic gradient, mean aortic gradient, and global ejection fraction. * Statistically significant.

	Cohort A	Cohort B	*p* Value
**Peak aortic gradient (mmHg)**			
baseline	85.7 ± 19.1	90.3 ± 25.7	0.272
discharge	20.9 ± 8.9	21.1 ± 10.8	0.635
30-day follow-up	20.4 ± 8.8	19 ± 9.2	0.429
1-year follow-up	20.6 ± 8.8	20.4 ± 9.8	0.893
2-year follow-up	21.7 ± 11.6	20.2 ± 8.8	0.478
3-year follow-up	22.4 ± 12.7	18.7 ± 9.8	0.130
**Mean aortic gradient (mmHg)**			
baseline	51.6 ± 12.6	52.7 ± 16.5	0.662
discharge	10.3 ± 5.5	10 ± 5.4	0.778
30-day follow-up	10.3 ± 5.1	10 ± 5.2	0.745
1-year follow-up	10.6 ± 5.3	10.6 ± 5.7	0.960
2-year follow-up	10.9 ± 5.7	10.4 ± 5.2	0.641
3-year follow-up	11.4 ± 7.7	9.3 ± 5.5	0.147
**Global ejection fraction (%)**			
baseline	55.2 ± 10.7	59.1 ± 10.8	0.051
discharge	58.9 ± 9.7	58.7 ± 8.7	0.901
30-day follow-up	58.3 ± 9	60 ± 9.1	0.306
1-year follow-up	57.2 ± 8.7	61 ± 7	0.015 *
2-year follow-up	57.6 ± 8.6	59.8 ± 8.7	0.229
3-year follow-up	58.8 ± 10.9	60.4 ± 10.6	0.499

**Table 8:** Echocardiographic parameters of the study population during the follow-up period.

*Transthoracic Echocardiography Follow-Up Data*						
Variable	Baseline (*n* = 122)	Discharge (*n* = 117, 95.9%)	30 Days (*n* = 117, 95.9%)	1 Year (*n* = 113, 92.6%)	2 Year (*n* = 104, 85.2%)	3 Year (*n* = 90, 73.8%)
Peak aortic gradient, mmHg	88.3 ± 22.9	20.5 ± 9.9	19.7 ± 8.9	20.5 ± 9.3	21 ± 10.3	20.6 ± 11.5
Mean aortic gradient, mmHg	52.4 ± 14.7	10.2 ± 5.5	10.2 ± 5.1	10.6 ± 5.5	10.6 ± 5.4	10.3 ± 6.8
LVEF, %	57 ± 11.1	58.8 ± 9.2	59.2 ± 9.1	59.1 ± 8.1	59.1 ± 8.4	59.8 ± 10.6
Aortic regurgitation grade 2 or above	8	10	9	10	19	11
Paravalvular leak grading mild/mild-to-moderate	NA	0/0	0/0	0/0	1/0	3/2
Mitral regurgitation grade 3 or 4	18	6	3	8	12	11

## Data Availability

The data presented in this study are available on request from the corresponding author. The data are not publicly available due to Hungarian legal regulations.

## Supplementary Materials

The following supporting information can be downloaded at: https://www.mdpi.com/article/10.3390/jcm13041088/s1, Table S1: Detailed data regarding the members of the TAVR team, role in the procedure and starting date of their profession; Table S2: Detailed data regarding the reason for in-hospital mortality; Table S3: Vascular complication of the study population based on the VARC-2 definition. AFC: common femoral artery. Reference [12] is cited in Supplementary Materials.

## Abbreviations

AS—aortic stenosis; TAVR—transcatheter aortic valve replacement; SAVR—surgical aortic valve replacement; NYHA—New York Heart Association; PPI—permanent pacemaker implantation; VARC-2—Valve Academic Research Consortium-2; TF—transfemoral access; THV—transcatheter heart valve; LFLG-AS—low-flow, low-gradient aortic stenosis; PLGLG-AS—paradox low-flow, low-gradient aortic stenosis.
