# Supplementary material for: Learning Curve for Starting a Successful Single-Centre TAVR Programme with Multiple Devices: Early and Mid-Term Follow-Up
Source: J Clin Med. 2024 Feb 14;13(4):1088. doi: 10.3390/jcm13041088 (PMC10889517; doi:10.3390/jcm13041088)
Supplement: Supplementary file 1 [file jcm-13-01088-s001.zip › jcm-2857689-supplementary.pdf]

Supplementum

S2.4.1 Transfemoral approach

After the 4 French (Fr) sheath was inserted, angiography from the femoral artery was performed (micro-puncture technique) to confirm the correct puncture site. After pre-dilatation of the femoral artery with 6 Fr sheath, two Proglide ((Abbott Vascular, CA, USA) sutures were inserted, and further pre-dilatation with 8-10-12 Fr sheaths was performed. Finally, the guiding sheath was inserted depending on the THV specification, or the in-line sheath technique was used when available.

S2.4.2 Trans-subclavian approach

This technique requires direct isolation of the left subclavian artery by a vascular surgeon. After the direct puncture of the artery, 6 Fr sheath was placed, and angiography was performed to confirm safe vascular access. A standard 0.035-inch guidewire was placed in the aortic root and exchanged for a pre-shaped 0.035-inch stiff wire through a pigtail catheter, and then a guiding sheath was inserted. The implantation of the THV is the same as in TF technique. Finally, the puncture site was closed surgically. Angiography from the left radial artery ruled out mechanic complications of the subclavian artery. For dissection or flow-limiting stenosis, percutaneous stent implantation was performed if needed.

S2.4.3 Trans-axillary approach

This technique was used in patients who were not suitable for TF approach. It was based on CT measurements confirming that the axillary artery had an adequate inner diameter. A vascular surgeon directly isolated the left axillary artery. Next, direct 6 Fr sheath insertion and

angiography were performed to prove the safe vascular access. After the standard 0.035-inch guidewire was placed in the aortic root, it was exchanged for a pre-shaped 0.035-inch stiff wire through a pigtail catheter, and then a guiding sheath was inserted. The implantation of the THV was the same as the TF technique, and the puncture site was closed surgically. Angiography from the left radial artery ruled out mechanic complications of the subclavian artery. For dissection or flow-limiting stenosis, percutaneous stent implantation was performed if needed.

S2.4.4 Direct aortic approach

This technique required a mini-sternotomy at the right second intercostal space. The cardiac surgeon prepared the ascending aorta. Next, aortography with a pigtail catheter was performed to measure the distance between the aortic annulus and the selected entry site in the ascending aorta. At the entry site, two aortic purse-string sutures for direct aortic access were placed in a standard fashion, as previously reported [12]. The 6 Fr sheath was inserted, and angiography was performed to confirm the optimal puncture site. Next, a standard 0.035-inch guidewire was placed into the aortic root and exchanged for a pre-shaped 0.035-inch stiff wire through a pigtail catheter. Guiding sheath insertion was performed through the stiff wire under direct and fluoroscopic visualisation. The implantation of the THV was the same as in the TF technique. Finally, the puncture site was closed surgically. Angiography from the left radial artery was used to rule out mechanic complications.

Supplementary Tables

Member of TAVR team	Role in the TAVR team	Form of the profesion	Starting date of the profession
IH	first operator	internist/cardiologist/interventional cardiologist/anaesthetist	1998
BM	second operator	cardiologist/interventional cardiologist/intensive therapeutist	2005
RK	anaesthetist	anaesthetist	2006
IG	cardiac sonographer	internist/cardiologist	1998
GK	vascular surgeon	surgeon/vascular surgeon	1995
LL	heart surgeon	heart surgeon	1995

Supplementary Table S1,

Detailed data regarding the members of the TAVR team, role in the procedure and starting date of their profession.

Patient number	Reason for in-hospital mortality
1	Pericardial tamponade - treated surgically. TAVR was implanted with severe aortic regurgitation (grade III). ViV was performed three days after but due to severe hemodynamic deterioration patient died.
2	TAVR was implanted via direct aortic approach successfully. Due to bilateral pleural fluidum percutaneous thoracocentesis was performed, resulted in haemothorax. Surgical intervention was imperative and successful. After one week, the patient died from multi-organ failure.
3	Two days after the post-TAVR PPI, pacemaker dysfunction occured, and immediate temporary pacemaker implantation was mandatory. After repositioning the permanent pacemaker electrodes, the temporary pacemaker electrode was removed, and a pericardiac tamponade occurred, which was resolved surgically. Three days after, due to multi-organ failure we lost the patient.
4	Severe sepsis due to pneumonia occured, which was treatment refractory. Multi-organ failure evolved and caused the death of the patient
5	Four days after TAVR, patient suffered from a haemorrhagic stroke leading to the death of the patient.

Supplementary Table S2,

Detailed data regarding the reason for in-hospital mortality.

Vascular complications of study population

**Patient No**	**Form of vascular complication**	**Intervention to treat**	Amount of transfusion (U)
1	minor	surgical intervention of AFC	2
2	minor	stent implantation of axillary artery	2
3	minor	surgical re-intervention of access site	2
4	major	stent implantation of AFC	6
5	minor	surgical intervention of AFC	0
6	minor	stent implantation of subclavian artery	0
7	minor	surgical intervention of left brachial artery pseudoaneurysm	2
8	minor	surgical intervention of AFC	0
9	major	balloon angioplasty of AFC	6
10	minor	surgical embolectomy of brachial artery	2
11	minor	stent implantation of AFC	2
12	minor	surgical intervention of AFC	0
13	minor	balloon angioplasty of AFC	0
14	major	surgical intervention of AFC	4
15	major	surgical intervention of AFC	4
16	minor	stent implantation of AFC	2
17	minor	balloon angioplasty of AFC	0
18	minor	balloon angioplasty of AFC	0

Supplementary Table S3,

Vascular complication of the study population based on the VARC-2 definition. AFC: common femoral artery.
